# CCR9^+^CD4^+^ T cells are associated with disease activity in patients with rheumatoid arthritis

**DOI:** 10.1097/MD.0000000000037803

**Published:** 2024-04-19

**Authors:** Geng Lina, Qiang Cuixin, Zhang Xia, Yang Jing, Li Zhirong, Ouyang Zirou, Li Jiayiren, Zhang Yulian, Huo Qiuyue, Li Qianqing, Liu Yan, Qin Pu, Zhang Bin, Zhao Jianhong

**Affiliations:** a Provincial Center for Clinical Laboratories, The Second Hospital of Hebei Medical University, Shijiazhuang, Hebei, China; b Department of Clinical Laboratory, The First Affiliated Hospital of Hebei North University, Zhangjiakou, Hebei, China; c Department of Rheumatology and Immunology, The First Affiliated Hospital of Hebei North University, Zhangjiakou, Hebei, China.

**Keywords:** CCR9^+^CD4^+^ T cells, chemokine receptor, rheumatoid arthritis, synovial fluid

## Abstract

An increase in CD4^+^ T cells in the synovium is closely linked to the pathogenesis of rheumatoid arthritis (RA). We aimed to identify the possible causes of the elevated CD4^+^ T cell levels and to explore the factors influencing disease activity in RA. Fifty-five RA patients, including 28 with active RA (ARA), 27 with inactive RA, and 22 healthy controls, were recruited for this study. The proportion of CCR9^+^CD4^+^ T cells and the expression of chemokine receptor 9 (CCR9) on CD4^+^ T cells were analyzed by flow cytometry. Enzyme-linked immunosorbent assay and chemiluminescent immunoassay were used to evaluate interleukin (IL)-17A and IL-6 levels, respectively. The proportion of CCR9^+^CD4^+^ T cells and the expression of CCR9 on CD4^+^ T cells increased significantly in peripheral blood (PB) and synovial fluid (SF) in ARA compared to those in inactive RA. Furthermore, SF contained more CCR9^+^CD4^+^ T cells, IL-6, and IL-17A than PB in RA patients. Moreover, CD4^+^ T cells in the PB of patients with RA, especially ARA, expressed more CCR9 and secreted more IL-6 and IL-17A after activation. Here, we also demonstrated that both the percentage of CCR9^+^ cells in CD4^+^ T cells and the expression of CCR9 on circulating CD4^+^ T cells were positively correlated with erythrocyte sedimentation rate, hypersensitive C-reactive protein, rheumatoid factor, and anti-cyclic citrullinated peptide antibody. CCR9^+^CD4^+^ T cells are elevated in PB and SF, and are associated with disease activity in patients with RA.

## 1. Introduction

Rheumatoid arthritis (RA) is an autoimmune disease characterized by joint destruction, systemic involvement, and presence of autoantibodies. The pathogenesis of RA is complex and involves a variety of immune cells. CD4^+^ T cells are believed to play a crucial role in RA, as they participate in the induction and propagation of inflammatory responses by secreting pro-inflammatory cytokines, growth factors, and interferons^[1]^ and stimulating the proliferation and differentiation of B cells.

Trafficking of CD4^+^ T cells and their inflammatory mediators into joints contributes to the initiation, propagation, and maintenance of synovial inflammation.^[2]^ The migration of CD4^+^ T cells depends on many factors, especially the expression of chemokines.^[3]^ It has been found that in RA patients, some chemokine receptors, such as CCR1,^[4]^ CCR7,^[5]^ and CXCR4^[2]^ on CD4^+^ T cells, bind to their ligands in synovium to facilitate the migration of CD4^+^ T cells to joints, while specific chemokine receptor antagonists can attenuate the chemokine effect.^[4]^ Chemokine receptor 9 (CCR9) belongs to the β-chemokine receptor family that binds to its specific ligand, chemokine ligand 25 (CCL25),^[6]^ and its signaling contributes to the development of mucosal immunity and intestinal inflammation through chemotaxis of T cells into the gut mucosa.^[7]^ In Crohn disease, the proportion of CCR9^+^CD4^+^ T cells and the expression of CCL25 in intestinal tissues are increased and correlated with inflammatory activity.^[8]^ Furthermore, the frequency of CCR9^+^CD4^+^ T cells in peripheral blood (PB) is increased in patients with gastrointestinal diseases such as functional dyspepsia,^[9]^ small bowel Crohn disease,^[10]^ and delayed gastric emptying.^[11]^ These findings reveal the importance of CCR9^+^CD4^+^ T cells in gastrointestinal disease; however, whether this population has a critical role in RA remains unclear. In the autoimmune disease primary Sjögren syndrome, enriched CCR9 expression on mucosal-associated invariant T cells may facilitate migration to inflamed salivary glands known to overexpress CCL25.^[12]^ In recent years, CCR9 and CCL25 have been found to be more abundant on monocytes/macrophages in the PB and synovium in RA.^[13–15]^ Furthermore, CCL25 may be involved in the differentiation of monocytes into macrophages, particularly in RA.^[13]^ In animal experiments, the inflammatory response in collagen-induced arthritis was suppressed in CCR9-deficient mice.^[14]^ Since CCL25 expression is increased in the synovium of RA patients, it may recruit more CCR9^+^CD4^+^ T cells to the joint. However, the role of CCR9^+^CD4^+^ T cells in PB and synovial fluid (SF) in RA remains unclear, and is the focus of the current investigation.

In the present study, our objective was to investigate the proportion of CCR9^+^CD4^+^ T cells in PB and SF, and to explore the effects of CCR9^+^CD4^+^ T cells on disease activity in RA.

## 2. Materials and methods

### 2.1. Study population

All 55 patients with RA and 22 age- and sex-matched healthy controls (HC) were recruited from The First Affiliated Hospital of Hebei North University. Individuals with RA were diagnosed according to the 2010 American College of Rheumatology/European League Against Rheumatism classification criteria for RA and classified according to the Disease Activity Score 28 (DAS28) based on erythrocyte sedimentation rate (ESR) (active RA [ARA], DAS28-ESR ≥ 2.6 units; inactive RA [IRA], DAS28-ESR < 2.6 units). The exclusion criteria were as follows: a diagnosis of known autoimmune diseases (for RA patients, the exclusion criteria were a diagnosis of known autoimmune diseases other than RA); a history of severe chronic infection, any current infection; and diagnosis of cancer.

Blood samples were obtained from patients with ARA (n = 28), IRA (n = 27), and HC (n = 22). SF samples were obtained from patients with ARA (n = 6), IRA (n = 5), and control samples (n = 5, diagnosed with osteoarthritis [OA], and the exclusion criteria were diagnosis of cancer, infection, and autoimmune diseases).^[16]^

The clinical and laboratory variables of the patients were recorded at the time of enrollment. Laboratory indicators associated with RA include ESR, hypersensitive C-reactive protein (hs-CRP), rheumatoid factor (RF), and anti-cyclic citrullinated peptide antibody (antiCCP). The study was approved by the Ethics Committee of The First Affiliated Hospital of Hebei North University. All participants have informed consent.

### 2.2. Cell isolation, culture, and stimulation

Peripheral blood mononuclear cells (PBMCs) and synovial fluid mononuclear cells (SFMCs) were isolated by density gradient centrifugation using the Ficoll-Hypaque solution. PBMCs were treated in 24-well culture plates (1 × 10^7^ cells/mL) with 1 μL/well Leukocyte Activation Cocktail (BD Biosciences, San Jose, CA) containing the phorbol ester, PMA, ionomycin, and Brefeldin A in a CO_2_ incubator at 37 °C for 5 hours, and then the cells and supernatant were collected. PBMCs treated under the same culture conditions without Leukocyte Activation Cocktail were used as the negative control group. All operations were performed according to the manufacturer’s instructions.

### 2.3. Flow cytometry analysis

PBMCs and SFMCs were washed and stained with BB515 Mouse Anti-Human CD4 antibody (clone RPA-T4; BD Biosciences) and Alexa Fluor 647 Mouse Anti-Human CCR9 (clone 112509; BD Biosciences) to detect the proportion of CCR9^+^CD4^+^ T cells and the expression of CCR9 on CD4^+^ T cells. Isotype-matched mAb controls were used for all procedures. The cells were analyzed using a flow cytometer (BD FACSCanto II; BD Biosciences). All operations were performed according to the manufacturer’s instructions.

### 2.4. Cytokine detection

Cytokine concentrations (interleukin (IL)-6 and IL-17A) in the PB, SF, and culture supernatants were measured. IL-17A levels were measured using enzyme-linked immunosorbent assay kits (Elabscience), and IL-6 levels were measured using chemiluminescent immunoassay (Wantai Biopharm). All operations were performed according to the manufacturer’s instructions.

### 2.5. Statistical analysis

Multigroup comparisons were performed using a one-way analysis of variance. Comparisons between 2 groups were performed using a 2-tailed Student *t* test. Comparisons of paired samples were performed using the Wilcoxon signed-rank test. Correlations were examined using the Spearman rank test. Data are expressed as means ± standard deviation (SD). A *P*-value of .05 or less was considered significant. All analyses were performed using GraphPad Prism 8 software (San Diego, CA).

## 3. Results

### 3.1. Clinical and laboratory characteristics in RA patients and HC

Fifty-five patients with RA (28 ARA and 27 IRA) and 22 HC were recruited for this study. No significant differences were found in sex (male: 21.4% vs 18.5% vs 22.7%, respectively) and age (54.17 ± 9.91 vs 53.88 ± 13.14 vs 50.05 ± 8.98 years, respectively) among the 3 groups. However, there were significant differences in ESR, hs-CRP, antiCCP, and RF levels among the groups. The most important clinical data and laboratory test indicators of patients are presented in Table 1.

**Table 1:** Clinical and laboratory characteristics in patients with rheumatoid arthritis and healthy control.

	ARA	IRA	HC	*P* value (one-way ANOVA)	Reference interval
Number	28	27	22	ns	/
Sex (M/F)	6/22	5/22	5/18	ns	/
Age (yr)	54.17 ± 9.91	53.88 ± 13.14	50.05 ± 8.98	ns	/
ESR (mm/h)	52.90 ± 30.44	23.57 ± 11.78	6.59 ± 2.70	<.001	0–15 (M)/20 (F)
hs-CRP (mg/L)	26.65 ± 18.60	7.02 ± 15.2	5.94 ± 2.73	<.001	0–10
antiCCP (U/mL)	187.10 ± 81.22	77.62 ± 55.83	1.90 ± 0.73	<.001	0–5
RF (IU/mL)	414.24 ± 757.08	94.78 ± 111.37	7.36 ± 2.97	.006	0–20

Data are shown as mean ± SD.

ANOVA = one-way analysis of variance, antiCCP = anticyclic citrullinated peptide, ARA = active rheumatoid arthritis, ESR = erythrocyte sedimentation rate, F = female, HC = healthy controls, hs-CRP = hypersensitive C-reaction protein, IRA = inactive rheumatoid arthritis, M = male, ns = not significant, RF = rheumatoid factor.

### 3.2. CCR9 expression on CD4^+^ T cells in PB of RA patients

**Figure 1.**
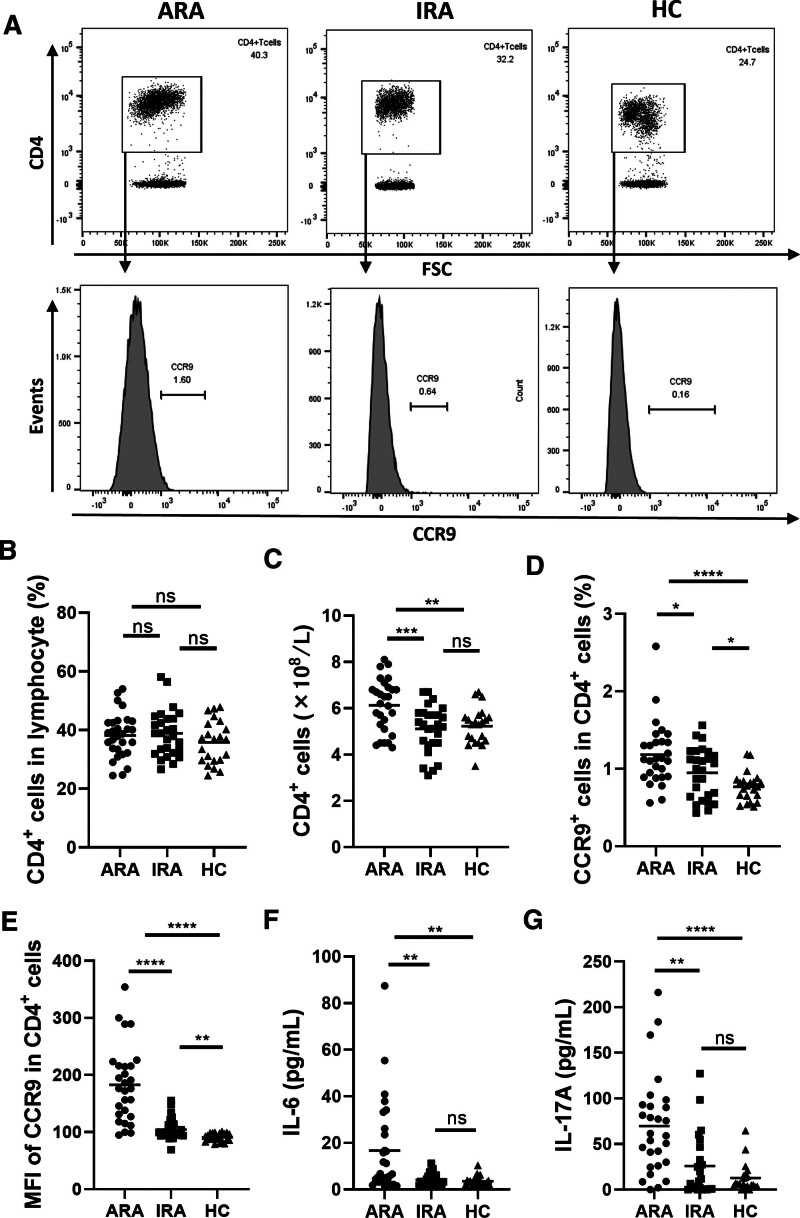
CCR9 expression on CD4^+^ T cells in PB of RA patients. (A) Representative plots of the percentage of CD4^+^ T cells in lymphocytes and CCR9 expression on CD4^+^ T cells. (B) The percentage of CD4^+^ T cells in lymphocyte, (C) the absolute numbers of CD4^+^ T cells, (D) the percentage of CCR9^+^ cells in CD4^+^ T cells, and (E) the MFI of CCR9 on circulating CD4^+^ T cells from RA patients and HC were analyzed by flow cytometry (FCM). (F) IL-6 was analyzed by chemiluminescent immunoassay. (G) IL-17A was analyzed by ELISA. Each data point represents an individual subject; horizontal lines show the mean. *P < .05; **P < .005; ***P < .0005; ****P < .0001; ns, not significant. ARA = active rheumatoid arthritis, CCR9 = chemokine receptor 9, HC = healthy controls, IL = interleukin, IRA = inactive rheumatoid arthritis, MFI = mean fluorescence intensity, PB = peripheral blood, RA = rheumatoid arthritis.

CD4^+^ T cells are involved in RA inflammation; therefore, we first examined the number of CD4^+^ T cells in the PB. Representative plots of the percentage of CD4^+^ T cells in lymphocytes and CCR9 expression on CD4^+^ T cells were shown in Figure 1A. The absolute number of CD4^+^ T cells (Fig. 1C), but not the percentage of CD4^+^ T cells (Fig. 1B), increased significantly in ARA compared to IRA (*P* = .021) and HC (*P* < .0001). In addition, the frequency of CCR9^+^CD4^+^ T cells was higher in ARA than in IRA (*P* = .024) and HC (*P* < .001), and was higher in IRA than in HC (*P* = .025) (Fig. 1D). Further, the expression of CCR9 on CD4^+^ T cells was significantly elevated in ARA compared to IRA (*P* = .024) and HC (*P* < .001), and was elevated in IRA compared to HC (*P* = .025) (Fig. 1E).

Since IL-6 and IL-17 are associated with the pathogenesis of RA and can be secreted by CD4^+^ T cells,^[17,18]^ we next measured the serum levels of these 2 cytokines. The levels of IL-6 and IL-17A were higher in ARA than IRA and HC, but no significant difference was found between IRA and HC (Fig. 1F and G).

### 3.3. CCR9 expression on CD4^+^ T cells in SF of RA patients

**Figure 2.**
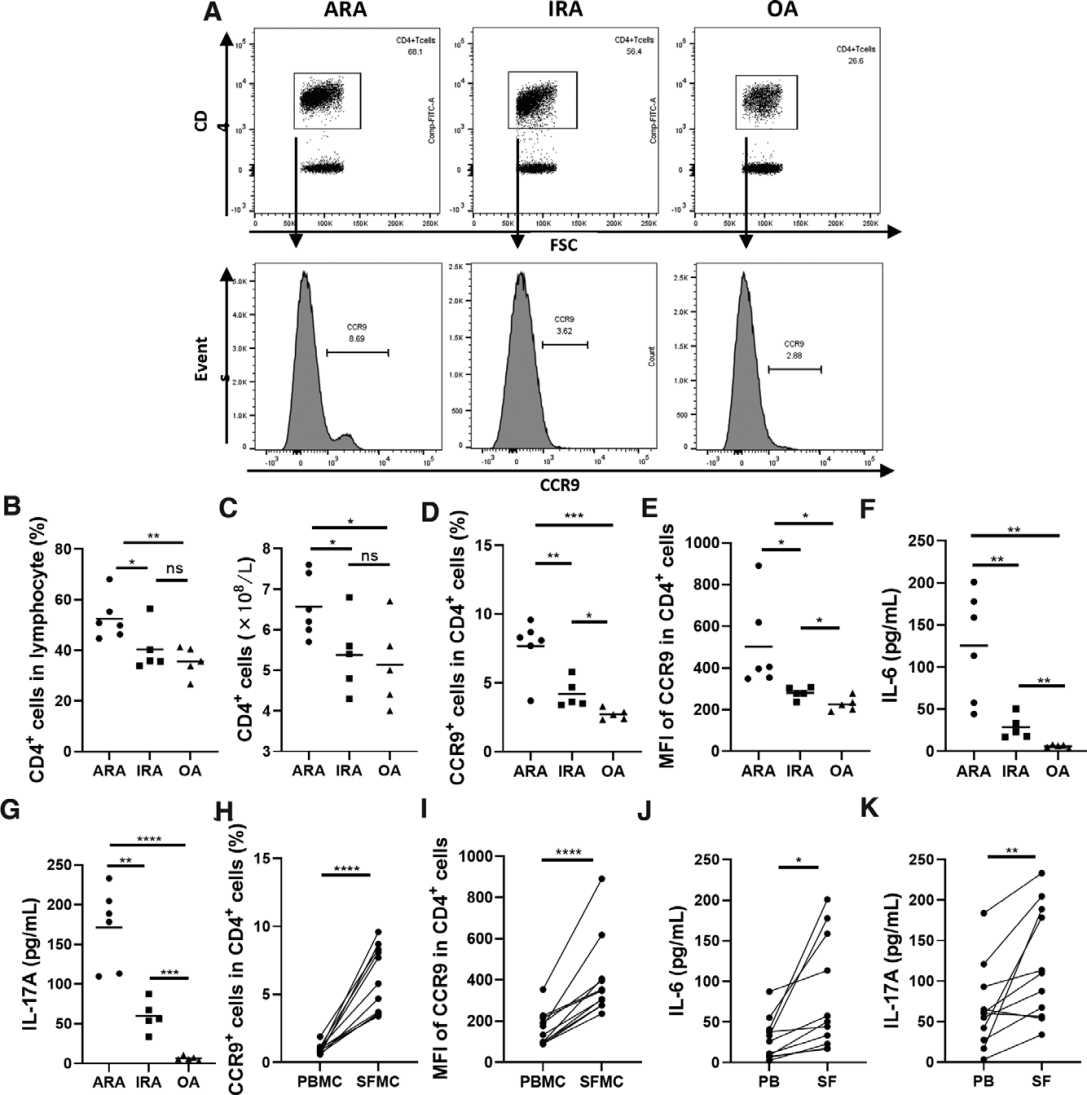
CCR9 expression on CD4^+^ T cells in SF of RA patients. (A) Representative plots of the percentage of CD4^+^ T cells in lymphocytes and CCR9 expression on CD4^+^ T cells. (B) The percentage of CD4^+^ T cells in lymphocyte, (C) the absolute numbers of CD4^+^ T cells, (D) the percentage of CCR9^+^ cells in CD4^+^ T cells, and (E) the MFI of CCR9 on circulating CD4^+^ T cells were analyzed by FCM. Levels of (F) IL-6 and (G) IL-17A in SF were analyzed. The differences of (H) the percentage of CCR9^+^ cells in CD4^+^ T cells, (I) the MFI of CCR9 on circulating CD4^+^ T cells, (J) IL-6, and (K) IL-17A between PB and SF are shown. Each data point represents an individual subject; horizontal lines show the mean. *P < .05; **P < .005; ***P < .0005; ****P < .0001; ns, not significant. ARA = active rheumatoid arthritis, CCR9 = chemokine receptor 9, FCM = flow cytometry, IL = interleukin, IRA = inactive rheumatoid arthritis, MFI = mean fluorescence intensity, OA = osteoarthritis, PB = peripheral blood, RA = rheumatoid arthritis, SF = synovial fluid.

Since RA inflammation occurs mainly in the joints, we next explored CCR9 expression on CD4^+^ T cells in SFMC. Representative plots of the percentage of CD4^+^ T cells in lymphocytes and CCR9 expression on CD4^+^ T cells were shown in Figure 2A. Both the percentage of CD4^+^ T cells (Fig. 2B) and the absolute number of CD4^+^ T cells (Fig. 2C) increased significantly in ARA compared with IRA and OA, but there was no difference between IRA and OA (Fig. 2D). Consistent with PBMC, the frequency of CCR9^+^CD4^+^ T cells was higher in ARA than in IRA (*P* = .007) and OA (*P* = .0005), and was higher in IRA than in OA (*P* = .017) in SFMC (Fig. 2C). Furthermore, CCR9 expression in CD4^+^ T cells was elevated in ARA compared with IRA (*P* = .049) and OA (*P* = .020), and was elevated in IRA compared with OA (*P* = .024) (Fig. 2E). As expected, the levels of both IL-6 (Fig. 2F) and IL-17A (Fig. 2G) were higher in ARA than in IRA and OA, and were higher in IRA than in OA in SF. In addition, the percentage of CCR9^+^ CD4^+^ T cells (Fig. 2H), the mean fluorescence intensity (MFI) of CCR9 on CD4^+^ T cells (Fig. 2I), IL-6 (Fig. 2J), and IL-17A (Fig. 2K) levels were all elevated in SF compared to PB.

### 3.4. CCR9 expression on CD4^+^ T cells after activation in vitro

**Figure 3.**
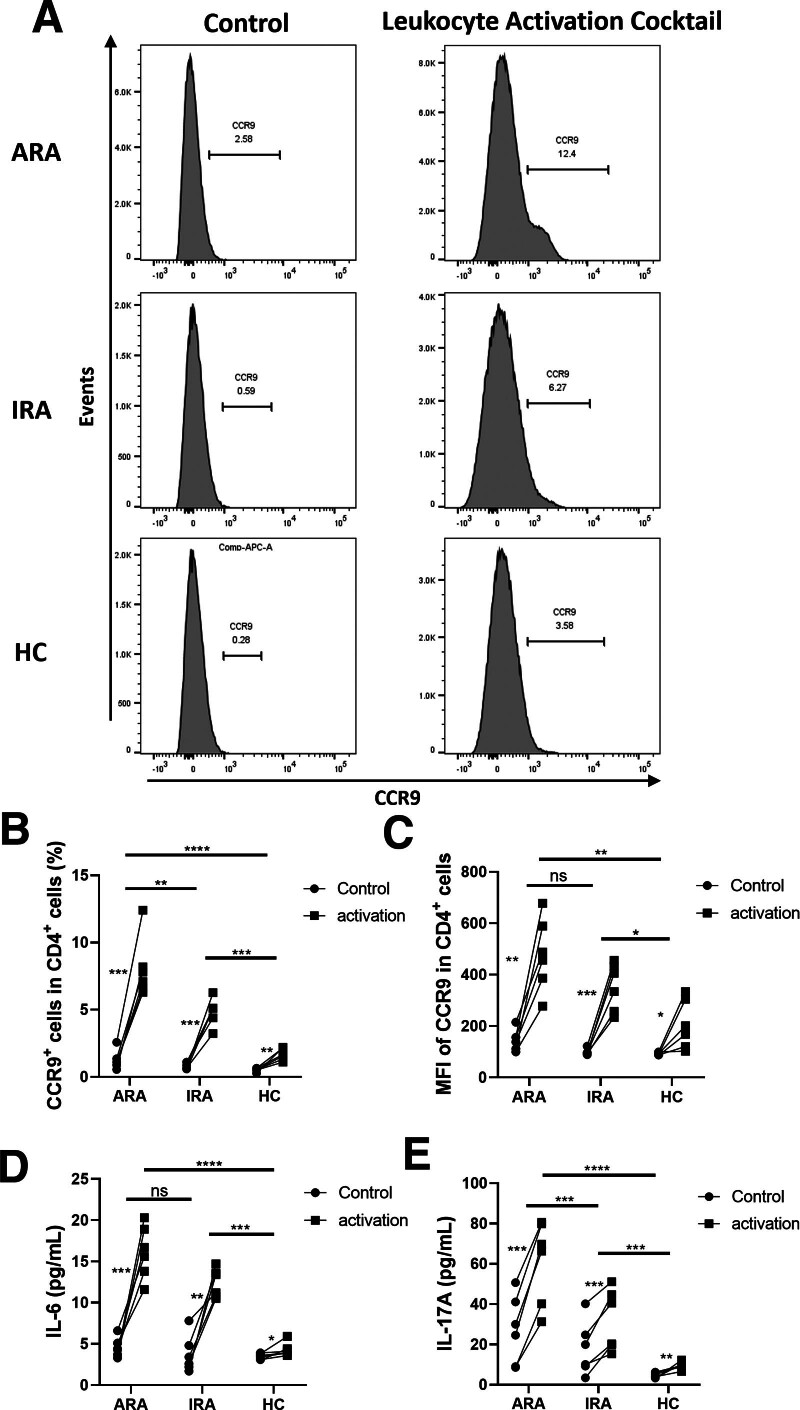
CCR9 expression on CD4^+^ T cells after activation in vitro. (A) Representative plots of CCR9 expression on CD4^+^ T cells in PB treated with or without Leukocyte Activation Cocktail from the RA patients and HC. After activation, (B) the percentage of CCR9^+^ cells in CD4^+^ T cells, (C) the MFI of CCR9 on circulating CD4^+^ T cells, (D) IL-6, and (E) IL-17A in culture supernatant were analyzed. Each data point represents an individual subject; horizontal lines show the mean. *P < .05; **P < .005; ***P < .0005; ****P < .0001; ns, not significant. ARA = active rheumatoid arthritis, CCR9 = chemokine receptor 9, HC = healthy controls, IL = interleukin, IRA = inactive rheumatoid arthritis, MFI = mean fluorescence intensity, PB = peripheral blood, RA = rheumatoid arthritis.

As the frequency of CCR9^+^CD4^+^ T cells and the expression of CCR9 on CD4^+^ T cells were higher in SF than in PB, and most CD4^+^ T cells in PB are in a resting state, it is necessary to determine whether activation of CD4^+^ T cells leads to a different distribution of CCR9^+^CD4^+^ T cells between SF and PB. Hence, after isolating PBMC from ARA, IRA, and HC (6 cases in each group), we activated the cells with Leukocyte Activation Cocktail and measured the expression of CCR9 on CD4^+^ T cells, IL-6, and IL-17A. As expected, the results showed that the percentage of CCR9^+^ CD4^+^ T cells and the MFI of CCR9 on circulating CD4^+^ T cells, IL-6, and IL-17 in the culture supernatant were all elevated after activation of PBMC, and these changes were more prominent in ARA and IRA (Fig. 3). These results indicate that CD4^+^ T cells in the PB of RA patients, especially ARA, could express more CCR9 and secrete more inflammatory cytokines after activation.

### 3.5. The correlations between circulating CCR9^+^CD4^+^ T cells and disease activity

**Figure 4.**
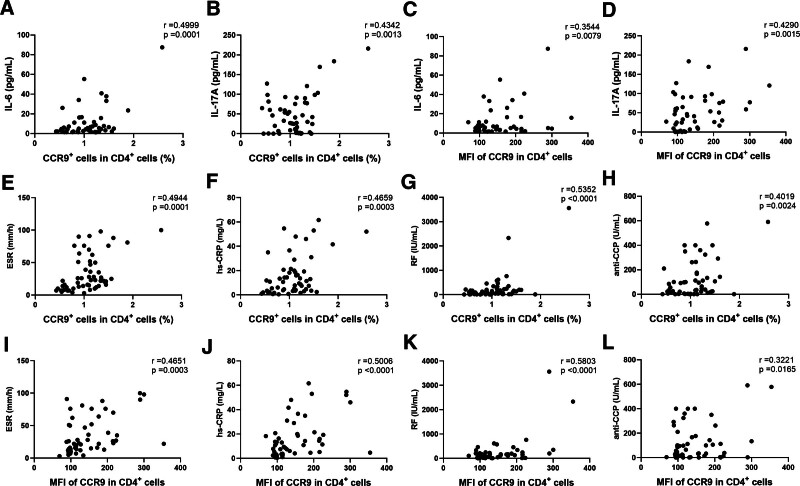
Correlations between CCR9^+^CD4^+^ T cells and disease activity. Correlations between the percentage of CCR9^+^ cells in CD4^+^ T cells and (A) IL-6, (B) IL-17A, (E) ESR, (F) hs-CRP, (G) RF, and (H) antiCCP. Correlations between the MFI of CCR9 on circulating CD4^+^ T cells and (C) IL-6, (D) IL-17A, (I) ESR, (J) hs-CRP, (K) RF, and (L) antiCCP. antiCCP = anti-cyclic citrullinated peptide antibody, CCR9 = chemokine receptor 9, ESR = erythrocyte sedimentation rate, hs-CRP = hypersensitive C-reactive protein, IL = interleukin, MFI = mean fluorescence intensity, RF = rheumatoid factor.

ESR, hs-CRP, RF, and antiCCP are critical indicators that provide evidence for the diagnosis and disease activity of RA; therefore, we analyzed the relationship between CCR9^+^CD4^+^ T cells in PB and the levels of these indicators in patients with RA. There were positive correlations between the percentage of CCR9^+^ CD4^+^ T cells, IL-6, and IL-17A (Fig. 4A and B). Similar results were observed for the MFI of CCR9 on circulating CD4^+^ T cells (Fig. 4C and D). In addition, both the percentage of CCR9^+^ cells and the MFI of CCR9 on circulating CD4^+^ T cells were positively correlated with ESR, hs-CRP, RF, and antiCCP (Fig. 4E–L). Collectively, these findings indicate that circulating CCR9^+^CD4^+^ T cells are associated with disease activity in RA patients.

## 4. Discussion

In RA, CD4^+^ T cells play a crucial role in inflammatory responses affecting the synovial tissue of joints by producing inflammatory cytokines and assisting B cells to produce antibodies.^[1,19]^ CD4^+^ T cells preferentially accumulate in a perivascular distribution and are elevated in the synovium in RA,^[20]^ partly due to the interaction of chemokines and chemokine receptors.^[3,5]^ Therefore, it is necessary to determine whether the disease activity of RA and inflammatory cytokines are associated with the expression of certain chemokine receptors on CD4^+^ T cells.

It is to be thought that CCR9 is constitutively expressed on T lymphocytes in the small intestine, thymus, lymph node, and spleen^[21]^; however, it has also been found in other parts of the body, such as cerebrospinal fluid^[22]^ and SF^[14]^ in recent years. Various evidences point to the chemotactic role of CCR9 and its specific ligand, CCL25. In patients with Sjögren syndrome, CCR9^+^CD4^+^ T cells are expanded in PB, and CCR9: CCL25 interactions induce the migration of CCR9^+^CD4^+^ T cells, which have a greater potential to provide IL-21.^[23]^ In another study of Sjögren syndrome, enriched CCR9 expression on mucosal-associated invariant T cells may facilitate migration to inflamed salivary glands known to overexpress CCL25.^[12]^ In this study, the percentage of CCR9^+^CD4^+^ T cells and the MFI of CCR9 on CD4^+^ T cells were all elevated in SF compared to PB, which indicated that more CCR9^+^CD4^+^ T cells had migrated to the joint in RA. In addition, CCR9^+^CD4^+^ T cells were elevated in ARA than IRA in PB and SF, which suggested a potential relationship between CCR9^+^CD4^+^ T cells and disease activity of RA.

This study showed that although CCR9^+^CD4^+^ T cells were increased in the PB of RA patients, the proportion of CCR9^+^ cells in CD4^+^ T cells was very low (0.54 - 2.58%), which was consistent with the findings of Zhang et al.^[24]^ They found that normal CD4^+^ T cells rarely express CCR9, whereas T-cell lineage lymphocytic leukemia CD4^+^ T cells highly express CCR9. In the present study, the proportion of CCR9^+^CD4^+^ T cells in SF was much higher than that in PB in the same RA patient, so what factor caused this difference in distribution? A previous study reported that CCR9 was upregulated by conventional CD4^+^ T cells after activation in intestinal lymphoid tissues.^[22]^ Consistent with this, the present study showed that not only the percentage of CCR9^+^CD4^+^ T cells but also the MFI of CCR9 on circulating CD4^+^ T cells was elevated after the activation of circulating CD4^+^ T cells. These results indicate that CCR9 is upregulated after the circulating CD4^+^ T cells in RA receive activation signals, which makes more CD4^+^ T cells have a greater ability to migrate to the joints.

In addition to their role in lymphocyte trafficking, chemokine receptors have been found to regulate gene expression in target cells, control cell proliferation, and cytokines production.^[25]^ In this study, CCR9^+^CD4^+^ T cells appeared to have pro-inflammatory effects because they were positively correlated with IL-6 and IL-17A. Since IL-17A is secreted mainly by T helper (Th) 17 cells, so what are the possible correlation between CCR9^+^CD4^+^ T cells and Th17 cells? One study found that the frequency of CCR9^+^ cells is significantly elevated in mesenteric lymph nodes, which leads to potentiated Th1 cell and Th17 cell differentiation.^[26]^ Similarly, TLR4^−/−^ mice were resistant to experimental autoimmune encephalomyelitis, which was attributed to reduced levels of CCL25 and decreased CCR9^+^CD4^+^ T cells in the spinal cord that contributed to diminished Th17 cell development.^[27]^ Furthermore, the elevated circulating CCR9^+^ T cells in patients with small bowel inflammation are responsible for enhanced interferon-γ and IL-17 production from mesenteric lymph nodes in patients with Crohn disease.^[10,28]^ The above research shows that CCR9^+^CD4^+^ T cells could promote Th17 cells differentiation, development, and IL-17 production. However, the exact correlation between CCR9^+^CD4^+^ T cells and Th17 cells in RA remains to be further explored. On the other hand, a study found that CCR9 expression on Tregs from spleen and draining lymph nodes of either normal or collagen-induced arthritis mice was undetectable.^[29]^ Another study indicated that binding of CCR9 to CCL25 inhibits the differentiation of CD4^+^ T cells differentiating to Tregs in vitro.^[30]^ Consistent with these results, this study showed positively correlated between CCR9^+^CD4^+^ T cells and IL-6, IL-17A as well as disease activity, which provide more clarity on the pro-inflammatory role of CCR9^+^CD4^+^ T cells in RA.

Since the current study showed the important role of CCR9^+^CD4^+^ T cells in the progression of RA, it might be a potential therapeutic target to treat RA. In a collagen-induced arthritis model, inflammation was suppressed in CCR9-deficient mice or using the CCR9 antagonist CCX8037.^[14]^ In contrast, in an antigen-induced arthritis model, CCR9-deficient mice developed arthritis that was not significantly different in severity from wild-type animals.^[21]^ These different results may be due to the different mouse models used, and the exact mechanism needs to be further explored.

This study has some limitations. First, as this study was a single-center study, the demographic and geographic homogeneity limits generalizability of the findings to diverse racial/ethnic groups, and multi-center study with other populations will be conducted to avoid the limitation; second, mechanisms of CCR9^+^CD4^+^ T cells influence on disease activity in patients with RA is unclear, and additional functional studies of patient CCR9^+^CD4^+^ T cells will be explored in future studies; third, sample collection is challenging in the clinical setting, so a large enough sample of SF were difficult to obtain.

In conclusion, the present study suggests that CCR9 expression on CD4^+^ T cells is elevated in PB and SF of RA patients, and CCR9^+^CD4^+^ T cells are associated with disease activity and pro-inflammatory cytokines. These results indicate that targeting CCR9^+^CD4^+^ T cells for therapeutic intervention could potentially lead to reduced inflammation and disease progression in RA.

## Author contributions

**Conceptualization:** Geng Lina, Zhao Jianhong.

**Data curation:** Qiang Cuixin, Zhang Yulian.

**Formal analysis:** Qiang Cuixin, Huo Qiuyue.

**Funding acquisition:** Geng Lina.

**Investigation:** Geng Lina, Yang Jing.

**Methodology:** Yang Jing, Li Qianqing, Ouyang Zirou.

**Project administration:** Zhao Jianhong.

**Resources:** Zhang Xia.

**Software:** Qin Pu.

**Supervision:** Zhang Bin.

**Validation:** Li Zhirong, Li Jiayiren.

**Visualization:** Liu Yan.

**Writing – original draft:** Geng Lina.

**Writing – review & editing:** Zhao Jianhong.
